# Further validation of the Japanese version of the Multidimensional Assessment of Interoceptive Awareness

**DOI:** 10.1186/s13104-019-4556-x

**Published:** 2019-08-20

**Authors:** Haruo Fujino

**Affiliations:** 1 Department of Special Needs Education, Oita University, 700 Dannoharu, Oita, 870-1192 Japan; 2 Graduate School of Human Sciences, Osaka University, Suita, Japan

**Keywords:** Body awareness, Body-mind, Interoceptive awareness, Validation studies, Interoception

## Abstract

**Objective:**

The Multidimensional Assessment of Interoceptive Awareness (MAIA) is a validated measure to assess interoceptive awareness. Although an earlier study evaluated the Japanese version of the MAIA, it did not examine the measure’s test–retest reliability and the data fit of the factor structure. This study aims to further validate the Japanese version of the MAIA.

**Results:**

In this cross-sectional study, 268 Japanese individuals participated. They completed the Japanese version of the MAIA and concurrent validity measures. The test–retest reliability of the Japanese version’s subscales ranged from adequate to high (intra-class coefficients = 0.76–0.85). Confirmatory factor analysis demonstrated that the Japanese six-factor structure had a good fit in Japanese. The MAIA subscales were moderately associated with scores on the Body Awareness Scale (rho = 0.25–0.49). The results indicated high test–retest reliability and further confirmed the validity of the six-factor structure of the Japanese version of the MAIA. Hence, the Japanese version of the MAIA is a useful measure to assess interoceptive awareness in the Japanese population.

**Electronic supplementary material:**

The online version of this article (10.1186/s13104-019-4556-x) contains supplementary material, which is available to authorized users.

## Introduction

Recent studies have focused on interoceptive awareness as an important factor affecting the adaptivity of psychological functioning. Interoception and body awareness are cardinal features of the therapeutic process in body—mind therapies and body-oriented psychotherapy [1–6]. Interoception is a multifaceted construct that includes interoceptive awareness, attention, detection, accuracy (sensitivity), and sensibility [7–12], whereas body awareness represents perceptual and conscious awareness of subjective physical experience [12]. There is substantial overlap between interoception and body awareness. Several studies have provided conceptual models that dysfunction in interoceptive processing; these are linked to mental disorders, suggesting the interoception’s mediating role in psychiatric and neurodevelopmental disorders [10, 13–15]. Two clinical studies suggested that interoceptive awareness mediates therapeutic changes in mindfulness interventions [16, 17]; however, further research is required to clarify interoception’s adaptive role in mental health and mind–body interventions. Accordingly, a validated multidimensional measure is required to investigate the different aspects of interoceptive awareness.

Although several self-report measures to assess interoceptive and/or body awareness are available, they assess only very limited aspects (e.g., negative aspects) of the concept, which may not capture the complex nature of interoceptive awareness [12]. The Multidimensional Assessment of Interoceptive Awareness (MAIA) questionnaire is designed to assess interoceptive awareness [18]. It has a multidimensional construct (eight dimensions) that reflects the multidimensional nature of interoceptive awareness, as suggested by earlier studies [12, 19]. Additionally, the MAIA has been used to assess changes in interoceptive awareness in interventional studies [17, 20].

Originally, the MAIA was developed in English; subsequently, translated versions were developed in many languages (https:/osher.ucsf.edu/maia/). An evaluation of the validity and reliability of the MAIA’s Japanese version using exploratory factor analysis demonstrated that the MAIA’s original factor structure was not replicated in a Japanese sample [21]. Shoji and colleagues suggested that the MAIA’s Japanese version has a six-factor structure, with several items loaded on other factors unlike the original structure [21]; however, the model fit of the six-factor and original eight-factor structures has not been examined for the MAIA’s Japanese version. Additionally, the test–retest reliability, which was not examined by the previous validation study, should be confirmed with the longitudinal evaluation of interoceptive awareness. Hence, further validation is required to evaluate the psychometric properties of the Japanese version. Accordingly, this study aims to examine the validity of a six-factor model of the MAIA in a Japanese sample.

## Main text

### Methods

#### Participants

In this study, 268 participants aged 18–27 years (mean age = 19.6; standard deviation, SD = 1.3) were recruited. Most of them were students at three institutions in Japan (Oita University, Osaka University, and Hiramatsu Oita College of Rehabilitation) from January 2017. Approximately half the total number of participants comprised women (n = 141, 53%). In addition to the aforementioned 268, 78 participants aged 18–31 years (mean age = 19.0, SD = 1.8) were recruited to examine test–retest reliability. Seventy-eight participants completed the MAIA 2 weeks (± 1 week) after the first evaluation. The minimum number of participants required for this study was determined using the COnsensus-based Standards for the selection of health Measurement Instruments (COSMIN) checklist criteria (7 × 32 [number of items]) [22].

All the participants were notified of the study’s purpose and methods, and assured that their privacy would be protected. Once they agreed to participate by completing a written consent form, they were administered the questionnaires. Subsequently, on receiving the completed questionnaires, the research assistant checked them for missing values.

This study was approved by the Oita University Faculty of Education Research Ethics Committee (28-009). Further, it was conducted in accordance with the World Medical Association’s Declaration of Helsinki.

#### Measures

##### Multidimensional Assessment of Interoceptive Awareness *(MAIA)*

The MAIA is a 32-item self-report questionnaire that assesses the eight dimensions of interoceptive awareness on a 6-point Likert scale (0 [never] to 5 [always]) [18]. The MAIA comprises the following eight independent domains of interoceptive awareness: “Noticing,” the awareness of one’s physical sensations (four items); “Not-Distracting,” the tendency to not use distraction to avoid discomfort (three items); “Not-Worrying,” the tendency to avoid worrying about uncomfortable physical sensations (three items); “Attention regulation,” the ability to control one’s attention to inner bodily sensations (seven items); “Emotional awareness,” the ability to understand specific body sensations as physical responses to emotions (five items); “Self-regulation,” the ability to regulate psychological distress using mindful attention to inner bodily sensations (four items); “Body listening,” the tendency to actively listen to the body to gain insight (three items); and “Trusting,” the experience of one’s body as safe and trustworthy (three items). The score for each scale ranges from 0 to 5, which is calculated by averaging the scores of the scale’s individual items. Higher scores indicate higher ability or tendency in each domain. Finally, the study used the MAIA’s Japanese-translated version [21].

#### Body Awareness Scale

The Body Awareness Scale (BAS) is a 20-item self-report measure that assesses body awareness and experience of physical sensations; it comprises four subscales [23]. We used two of its subscales, “Awareness of Bodily Feeling” and “Actual Bodily Feeling,” to confirm the MAIA’s concurrent validity. Whereas the first subscale assesses the tendency of being aware of inner physical sensations, the second assesses the ability to feel one’s physical feelings as they truly are. The Cronbach’s alpha values for the current sample’s subscales were 0.78 (Awareness of Bodily Feeling) and 0.65 (Actual Bodily Feeling).

#### Statistical analysis

We examined the internal consistency of the MAIA’s Japanese version using item–scale correlation and Cronbach’s alpha calculation. A Cronbach’s alpha higher than 0.70 indicated good internal consistency. Further, we evaluated the MAIA’s test–retest reliability by using an intra-class correlation coefficient (ICC) with a two-way, mixed-effects model. An ICC higher than 0.70 indicated good test–retest reliability.

Subsequently, using confirmatory factor analysis, we examined the model fit of the two models for the MAIA. The first model was the original eight-factor model that used all the 32 items of the MAIA, and the second model was the Japanese six-factor model that used the 25 items of the MAIA suggested by Shoji and colleagues [21]. The model fit was evaluated using Chi square statistics, the comparative fit index (CFI), the Tucker-Lewis Index (TLI), the standardized root mean square residual (SRMR), and the root mean square error of approximation (RMSEA). We considered nonsignificant Chi square statistics, CFI and TLI ≥ 0.95, and SRMR and RMSEA ≤ 0.08 to indicate a good fit. Further, we estimated the MAIA’s factor structure using a polychoric correlation matrix and the diagonally weighted least squares method, which is designed for ordinal data [24].

Additionally, we evaluated concurrent validity by considering associations between related concepts. The earlier validation study used the Five Facet Mindfulness Questionnaire, Difficulties in Emotion Regulation Scale, and Pain Catastrophizing Scale as concurrent measures [21]. In this study, we examined the association between the MAIA subscales and body awareness, which was not examined in the previous study. Corresponding scales were determined according to the measured concept in each domain prior to the analysis (Additional file 1: Table S1).

Statistical analyses were performed using R 3.4.1 statistical software (R Core Team, Vienna, Austria). The significance level was set at a two-tailed *p* < 0.05 after Bonferroni correction to achieve an overall family-wise error rate of 0.05 for the associations between the MAIA and the BAS subscales (12 tests). The corrected *p* values are reported in “Results” section.

### Results

#### Descriptive statistics for the MAIA items and reliability

Seven of the eight subscales adequately satisfied the criteria for high internal consistency and test–retest reliability. The item-total correlations were adequate in all subscales (0.70–0.88). Further, the Cronbach’s alphas values of all the MAIA subscales except one were high, ranging from 0.72 (Not-Distracting) to 0.87 (Attention Regulation) (Table 1). Similarly, the ICC of each subscale was high (0.74–0.87). However, the Not-Worrying subscale, which was included in the original structure, had poor internal consistency (α = 0.32) and slightly less test–retest reliability (ICC = 0.68).

**Table 1:** Descriptive statistics, internal consistency indices, and test–retest reliability of the MAIA subscales

Subscale	No. of items	Median	Mean	SD	Item-total correlation	Cronbach’s alpha	ICC (95% CI)
Original 8-factor structure							
Noticing	4	2.8	2.69	0.98	0.71–0.79	0.74	0.74 (0.63–0.83)
Not-distracting	3	2.7	2.64	1.00	0.74–0.82	0.72	0.76 (0.65–0.84)
Not-worrying	3	2.0	2.19	0.87	0.47–0.74	0.32	0.68 (0.54–0.78)
Attention regulation	7	2.6	2.49	0.85	0.70–0.83	0.87	0.85 (0.77–0.90)
Emotional awareness	5	2.8	2.70	1.00	0.72–0.87	0.85	0.87 (0.80–0.91)
Self-regulation	4	2.8	2.76	0.89	0.73–0.81	0.74	0.86 (0.79–0.91)
Body listening	3	2.0	2.13	1.04	0.85–0.86	0.82	0.81 (0.71–0.87)
Trusting	3	3.0	2.83	1.01	0.79–0.88	0.80	0.78 (0.68–0.86)
Japanese 6-factor structure							
Noticing^a^	5^a^	2.6	2.66	0.94	0.70–0.79	0.78	0.85 (0.77–0.90)
Not = distracting	3	2.7	2.64	1.00	0.74–0.82	0.72	0.76 (0.65–0.84)
Attention regulation	7	2.6	2.49	0.85	0.70–0.83	0.87	0.85 (0.77–0.90)
Emotional awareness^a^	3^a^	3.0	2.81	1.12	0.86–0.88	0.84	0.82 (0.73–0.88)
Body listening^a^	4^a^	2.3	2.31	0.99	0.74–0.83	0.82	0.82 (0.73–0.88)
Trusting	3	3.0	2.83	1.01	0.79–0.88	0.80	0.78 (0.68–0.86)

^a^The items included in the subscale differ from the original

#### Model fit and concurrent validity of the Japanese MAIA

To evaluate model fit, the original eight-factor and Japanese six-factor constructs were evaluated using confirmatory factor analysis. The original eight-factor model showed generally acceptable fit in all fit indices except RMSEA (0.084), whereas the six-factor model showed acceptable fit in all goodness-of-fit indices (CFI = 0.981, TLI = 0.978, SRMR = 0.067, RMSEA = 0.078 [90% confidence interval 0.071–0.085]) (Table 2). Further, the standardized coefficients of all the item loadings were sufficiently high (0.57–0.90).

**Table 2:** Fit indices from the confirmatory factor analysis of the Japanese MAIA

Model	Chi square	df	p	CFI	TLI	SRMR	RMSEA (90% CI)
Original 8-factor model (32 items)	1262.8	436	< 0.001	0.973	0.969	0.073	0.084 (0.079–0.090)
Japanese 6-factor model (25 items)	684.2	159	< 0.001	0.981	0.978	0.067	0.078 (0.071–0.085)

We examined the correlations between the six subscales of the Japanese MAIA and the BAS subscales. Most of the MAIA subscales were moderately correlated with the conceptually corresponding BAS subscales (Table 3). The Not-Distracting subscale was independent of the BAS scores. Finally, we confirmed 9 of the 10 predefined hypotheses on the correlations (Additional file 1: Table S1).

**Table 3:** Spearman’s correlation coefficients between the MAIA subscales (Japanese six-factor model) and the Body Awareness Scale

	Awareness of bodily	Actual bodily
	Feeling	Feeling
Noticing	0.49***	0.35***
Not-distracting	− 0.06***	0.10
Attention regulation	0.35***	0.44***
Emotional awareness	0.28***	0.25***
Body listening	0.35***	0.33***
Trusting	0.29***	0.44***

*** p < 0.001

### Discussion

This study evaluated the model fit of the original eight-factor and Japanese six-factor structures of the MAIA in a Japanese sample. The results conformed to the original Japanese validation study, suggesting a six-factor model fit for the Japanese MAIA and the potential necessity of modifying its original subscales.

The properties of the Japanese MAIA were similar to those of the original English and other translated versions, except for one subscale. Although seven of the eight subscales of the MAIA’s Japanese version showed good internal consistency and reliability, the Not-Worrying subscale had low internal consistency and slightly low test–retest reliability, which is in line with previous validation studies. These studies showed a low internal consistency for the Not-Distracting and Not-Worrying subscales [18, 20, 25–28]. In the current study, the Not-Distracting subscale had good internal consistency, whereas the Not-Worrying subscale showed poor internal consistency. This trend has been observed in clinical samples, as well (e.g., those with current or past low back pain or with eating disorders) [29, 30], suggesting psychometric issues in the construct.

In the confirmatory factor analysis, the Japanese six-factor model showed good fit to the current sample, whereas the original eight-factor model did not satisfy the good-fit criterion for RMSEA (≤ 0.08). The results suggest the necessity of making minor modifications (e.g., deletion or addition of items) to the original eight-factor model to validate the MAIA scale in cross-cultural contexts [20, 25, 26, 28]. Since an earlier study suggested that conceptual and cultural differences may affect the construct of the MAIA [21], the findings imply that subjective aspects of interoceptive awareness are affected by the conceptual framework of a culture or population.

The previous Japanese validation study showed moderate associations between the MAIA and concurrent measures [21]. In the current sample, we further demonstrated a moderate association between the MAIA and a body awareness measure. Although most of the pre-specified hypotheses were confirmed, the MAIA’s Not-Distracting subscale was not correlated with the BAS subscales. Accordingly, the concept measured by the Not-Distracting subscale may be independent compared to the other subscales [28, 31]. The original MAIA was recently updated due to the problems in some scales, as described in “Discussion” section. Currently, the updated 37-item MAIA version 2, which was unavailable at the time of this study’s data collection, is available [32]. Hence, the use of MAIA version 2 in future studies is recommended.

In conclusion, this study showed high test–retest reliability and reconfirmed the validity of the Japanese version of the MAIA’s six-factor structure. Hence, the Japanese version of the MAIA is a reliable and validated measure to assess interoceptive awareness in the Japanese population.

## Limitations

The study has several limitations. This study did not examine the associations between improvement in interoceptive awareness and other adaptive abilities or functioning of individuals. The study’s findings have limited applicability since the characteristics of the study’s sample are nearly identical to those of a sample used by an earlier study [21]. Additionally, the low Cronbach’s alpha for the BAS subscale Actual Bodily Feeling may limit the correlations that can be identified with the Not-distracting subscale of the MAIA.

## Additional file

**Additional file 1: Table S1.** Hypotheses tested for concurrent validity. The summary of the hypotheses tested for concurrent validity.

## Data Availability

The datasets used and analyzed during the current study are available from the corresponding author upon reasonable request to the corresponding author.

## Abbreviations

BAS: Body Awareness Scale
CFI: comparative fit index
COSMIN: COnsensus-based Standards for the selection of health Measurement Instruments
ICC: intraclass correlation coefficient
MAIA: Multidimensional Assessment of Interoceptive Awareness
RMSEA: root mean square error of approximation
SRMR: standardized root mean square residual
TLI: Tucker–Lewis Index
