# Supplementary material for: Further validation of the Japanese version of the Multidimensional Assessment of Interoceptive Awareness
Source: BMC Res Notes. 2019 Aug 20;12:530. doi: 10.1186/s13104-019-4556-x (PMC6701007; doi:10.1186/s13104-019-4556-x)
Supplement: Supplementary file 1 — Additional file 1: Table S1. Hypotheses tested for concurrent validity. The summary of the hypotheses tested for concurrent validity. [file 13104_2019_4556_MOESM1_ESM.docx]

| **Additional Table S1.** Hypotheses tested for concurrent validity. | |  |
| --- | --- | --- |
| **Associations between MAIA scales, FFMQ, and Body Awareness Scale** | | **Confirmed?** |
| 1) | MAIA Noticing will have moderate positive association (coefficient = 0.3–0.5) with the BAS Awareness of Bodily Feeling scale | Yes |
| 2) | MAIA Noticing will have moderate positive association (coefficient = 0.3–0.5) with the BAS Actual Bodily Feeling scale | Yes |
| 3) | MAIA Not Distracting will have weak positive association (coefficient = 0.1–0.3) with the BAS Awareness of Bodily Feeling scale | No |
| 4) | MAIA Attention Regulation will have moderate positive association (coefficient = 0.3–0.5) with the BAS Awareness of Bodily Feeling scale | Yes |
| 5) | MAIA Attention Regulation will have moderate positive association (coefficient = 0.3–0.5) with the BAS Actual Bodily Feeling scale | Yes |
| 6) | MAIA Emotional Awareness will have weak positive association (coefficient = 0.1–0.3) with the BAS Awareness of Bodily Feeling scale | Yes |
| 7) | MAIA Emotional Awareness will have weak positive association (coefficient = 0.1–0.3) with the BAS Actual Bodily Feeling scale | Yes |
| 8) | MAIA Body Listening will have moderate positive association (coefficient = 0.3–0.5) with the BAS Awareness of Bodily Feeling scale | Yes |
| 9) | MAIA Body Listening will have moderate positive association (coefficient = 0.3–0.5) with the BAS Awareness of Actual Bodily Feeling scale | Yes |
| 10) | MAIA Trusting will have moderate positive association (coefficient = 0.3–0.5) with the BAS Awareness of Actual Bodily Feeling scale | Yes |
